# Health and Psychological Predictors of Antibiotic Use in Infancy and Fathers’ Role

**DOI:** 10.3390/ejihpe15050066

**Published:** 2025-04-25

**Authors:** Marina Fuertes, Rita Almeida, Francisco Dionisio

**Affiliations:** 1 Center for Psychology of University of Porto (CPUP), 4200-135 Porto, Portugal; 2 Lisbon School of Education, Polytechnic Institute of Lisbon, 1549-003 Lisbon, Portugal; 3 Research Center for Psychological Science (CICPSI), Faculty of Psychology, University of Lisbon, 1649-013 Lisbon, Portugal; anaalmeida6@edu.ulisboa.pt; 4 cE3c—Centre for Ecology, Evolution and Environmental Changes & CHANGE—Global Change and Sustainability Institute, Faculty of Sciences, University of Lisbon, 1749-016 Lisbon, Portugal

**Keywords:** antibiotics, father–infant interactions, daycare attendance, paternal stress and health care involvement, father–infant attachment

## Abstract

Previous research has shown that antibiotic use during the first year is associated with infants’ difficult behavior, maternal low sensitivity, and infant insecure-ambivalent attachment. However, these results may depend on the extent and type of parental involvement, paternal stress related to infant care, or the infant’s exposure to infections. To explore this question, we analyzed the relationship between these factors and examined potential predictors of antibiotic use among demographic, health, and psychological variables. This study included 62 Portuguese infants and their fathers as participants. Demographic and health information was collected at birth, 3, 9, and 12 months. Father–infant interactive behavior was observed in free play at 3 months and infant attachment in Strange Situation at 12 months. Parental Stress and Parents’ Responsibility Scales were used at 9 and 12 months, respectively. Infants who received antibiotics in the first year were less cooperative, more difficult, and less passive in free-play interactions and were more likely to attend a center-based daycare than others. In this study, the predictors of antibiotic use are infant difficultness and daycare type.

## 1. Introduction

The discovery and development of antibiotics was a remarkable breakthrough of the 20th century. However, their overconsumption has several drawbacks. Antibiotics given to infants have been associated with obesity, asthma, and autoimmune diseases like type 1 diabetes (Mischke et al., 2018; Stewart et al., 2018; Stokholm et al., 2018). In the process of disrupting the normal development of gut microbiota, antibiotics eliminate essential bacteria that supports gut health, increasing the risk for diseases like Crohn’s (Geirnaert et al., 2017). Antibiotics can also harm brain development (Champagne-Jorgensen et al., 2019; Slykerman et al., 2017, 2019). Children who receive antibiotics are more likely to develop severe mental disorders later in life. There is also a connection between early antibiotic use and neurocognitive issues, including depression and anxiety, in childhood (Diamanti et al., 2022; Duong et al., 2022; Köhler et al., 2017; Köhler-Forsberg et al., 2019; Volker et al., 2022).

Antibiotic-resistance genes in clinical and environmental bacteria have evolved globally under human-driven selective pressures (Christaki et al., 2020; Perry et al., 2016). Besides targeting disease-causing bacteria, antibiotics also target non-pathogenic bacteria. Moreover, even non-pathogenic bacteria play a crucial role in the development of antibiotic resistance among pathogens (Dionisio et al., 2023a; Darmancier et al., 2022; Louca, 2025). As a result, bacteria resistant to available antibiotics are responsible for almost 1.3 million deaths annually (Murray et al., 2022).

An immediate solution to mitigate these disadvantages is to reduce antibiotic consumption. However, this practice may be challenging to implement because it may be difficult to determine when antibiotics are necessary. Nevertheless, over 30% of outpatient antibiotic prescriptions in 2010–2011 in the United States of America were deemed unnecessary (Fleming-Dutra et al., 2016). Antibiotic overprescription is also strongly suggested by comparing antibiotic consumption across countries. For instance, antibiotic consumption in Europe in 2021 was 125.0 mg/kg of biomass for humans (28 European Union/European Economic Area countries), ranging between 44.3 mg/kg for the Netherlands and almost four-fold more in France, 160.1 mg/kg (European Centre for Disease Prevention and Control (ECDC et al., 2024). Even within a single country, antibiotic consumption may vary considerably (L. Shallcross et al., 2017; L. J. Shallcross & Davies, 2014). As a result, a recent study found a strong correlation between antibiotic resistance in more than 300,000 bacterial genomes and antibiotic use across countries (Louca, 2025).

Cultural, sociological, and psychological causes can explain the variability of the overuse of antibiotics between countries (Deschepper et al., 2008; Dionisio et al., 2023b; Grigoryan et al., 2008; Krockow et al., 2022). For example, the likelihood of a patient receiving antibiotics rises with the number of past prescriptions they have had (Palin et al., 2019; L. Shallcross et al., 2017). Some studies suggest that prescribers often overestimate how much patients expect antibiotics and, to comply with patients’ expectations, tend to prescribe them (Macfarlane et al., 1997; Sirota et al., 2017). This dynamic involves psychological factors from both the patient and the prescriber. In another study involving nearly a thousand adults in Sweden, researchers examined the relationship between generalized trust (the confidence that most people can be trusted) and the willingness to delay antibiotic treatment. This involved situations where the health professional prescribes antibiotics but suggests waiting to see if the illness resolves itself. Additionally, the number of days most participants said they could tolerate postponing treatment affected an individual’s willingness to delay taking antibiotics (Rönnerstrand & Andersson Sundell, 2015). Furthermore, social contexts, such as day care centers, have been associated with increased antibiotic use, likely due to the higher incidence of respiratory and gastrointestinal infections among children in these settings (Rooshenas et al., 2014; Thrane et al., 2001).

Also, in pediatric visits, caregivers’ and infants’ behaviors seem to be associated with antibiotic use. Recent research has shown a link between the use of antibiotics in the first year of an infant’s life and the manifestation of challenging behavior, low maternal sensitivity, and insecure-ambivalent attachment (Fuertes et al., 2022a, 2022b; Fuertes et al., 2023b; Stern et al., 2020). Past research found that infants with an insecure-ambivalent attachment and difficult behavior overreact when facing stress and pain, which is particularly evident when infants are ill (Meredith & Strong, 2019; Pietromonaco & Powers, 2015). In previous works, we have conjectured that this could lead their mothers to seek antibiotics (Dionisio et al., 2023b; Fuertes et al., 2022b, 2023b).

As Bowlby first realized in his attachment theory, children who stay close to an attachment figure are more likely to receive care and protection when distressed or sick, increasing their chances of survival (Bowlby, 1969; Bretherton, 1992). Ainsworth found that infants use different strategies (secure, insecure-ambivalent, insecure-avoidant) to seek proximity when they feel distressed. Securely attached infants have caregivers who respond to their needs, while avoidant infants avoid contact with often unavailable caregivers. Ambivalent infants display inconsistent behavior, making them difficult to soothe. Finally, infants with avoidant attachment do not seek comfort from their caregiver when distressed (Ainsworth et al., 1978; Madigan et al., 2024).

Attachment security is key to emotional, cognitive, and health development (Andrews et al., 2011; Feeney, 2000; Meredith & Strong, 2019). Children with chronic illnesses often have insecure attachments, and those with insecure-avoidant or ambivalent attachment styles respond differently to pain and illness (Cassibba et al., 2004; Meredith & Strong, 2019; Pietromonaco & Powers, 2015; Simmons et al., 1995). Facing diseases and pain, children with an avoidant attachment externalize less and regulate their emotions better, while ambivalent children are more reactive, express more symptoms, and need more medical attention (Andrews et al., 2011; Feeney, 2000). Conversely, infants with secure attachment have caregivers who are responsive and sensitive to their needs. These caregivers help the infant manage emotions during stressful times, offer comfort, and foster a sense of safety. As a result, both infants and parents are better able to handle challenging situations, such as illness. Nevertheless, when parents experience high levels of parental stress and have difficulty managing it, they may be less able to provide adequate support to their children during illness (Deater-Deckard, 2004).

Fathers play a fundamental role in the education, health, and development of infants. This role can be both direct and indirect. Indirectly, for example, paternal involvement in the early days of a child’s life has been associated with improved maternal health outcomes—including reductions in postpartum depression—which, in turn, positively influence both the physical and mental health of the infant (Cabrera et al., 2014). Directly, greater paternal involvement has been linked to lower rates of asthma and obesity, as well as enhanced mental health and cognitive outcomes in children (Allport et al., 2018). In addition, previous research suggests that paternal factors, including attitudes and behaviors related to medication, are associated with infant antibiotic use, highlighting the need to consider fathers in research on early childhood health and health care practices. For example, a study conducted in the Netherlands involving 4250 fathers of children who did not receive antibiotics during the first five years of life and 1032 fathers of children who received antibiotics found that 9.1% of fathers in the latter group also used antibiotics, compared to 3.3% of fathers in the former group (Jong et al., 2012). Moreover, a study conducted in Brazil with 489 children (aged between 29 days and 18 years) and their parents found that antibiotic prescription in children increased with the mother’s age but not the father’s age. Intriguingly, antibiotic use was more likely among children whose fathers had completed 11 years of secondary education compared to those whose fathers had lower education levels, whereas maternal education was not associated with antibiotic prescription (Zhang et al., 2005).

Although a substantial body of research has focused on maternal behavior in relation to antibiotic use during infancy, studies that include fathers remain limited. Yet, considering that infant attachment to mothers and fathers develops independently, incorporating paternal behavior may yield novel insights and significantly enrich the existing body of knowledge. Indeed, an infant who is securely attached to their mother may still be insecurely attached to their father (De Wolff & van Ijzendoorn, 1997; Fuertes et al., 2016). Moreover, maternal and paternal interactive behaviors tend to differ, with research frequently describing fathers as more play-oriented partners, while mothers are more often associated with affective communication and caregiving routines (Faria et al., 2014; Lamb & Lewis, 2010; Paquette, 2004). Given that relationships function as dynamic systems, infants learn to adapt their behavior to the specific interaction styles of different caregivers and relational contexts (Tronick et al., 2020).

Caregivers and early relational experiences have profound and lasting effects on a person’s psychological, emotional, and physical well-being throughout their lifespan. Understanding the interface of health and infant relationships can lead to early interventions to improve mental and physical health outcomes.

### Aims

Our main goal in this study is to explore potential predictors of antibiotic use during infancy among interactive, relational, fathers’ stress, fathers’ involvement, and according to daycare attendance. Therefore, this experimental and longitudinal study addresses four specific aims:

Aim 1—To compare paternal and infant interactive behavior at 3 months in dyads where infants took antibiotics and dyads where infants did not take antibiotic. If infants with more sensitive mothers were less likely to take antibiotics, we expected the same result with fathers (Fuertes et al., 2022b; Stern et al., 2020).

Aim 2—To examine the prevalence of antibiotic use in relation to infant–father attachment patterns at 12 months. Previous studies with mothers have shown that ambivalently insecurely attached infants are more likely to receive antibiotic prescriptions, with significantly higher odds of antibiotic use compared to other infant (Fuertes et al., 2022a, 2022b). In this study, we expected to observe a similar effect with fathers.

Aim 3—To test possible contributors of antibiotic use in the first year of infants’ life.

Parents’ behavior is affected by their responsibilities (Fuertes et al., 2016) and ability to manage stress (Johansson et al., 2021). Thus, we intend to study paternal involvement in health care at 9 months and paternal stress at 12 months in dyads where infants took antibiotics and dyads where infants did not take. We expected that more involved and stressed parents would be more eager to help and find a solution to alleviate their children’s symptoms, which may impact antibiotic use.

Besides infant and father factors, other factors can account for antibiotics consumption. As pointed in the literature revisions, several factors account for antibiotic use, namely the exposition to infections. Since infants attending daycare centers may have increased exposure to infectious agents (Collet et al., 1991), we expected that a longer duration in center-based daycare would be associated with a higher likelihood of antibiotic use.

Aim 4—To identify predictors of antibiotic use among the factors found in aim 1, 2, or 3. We expect that paternal and infant interactive behavior and the quality of infant attachment, based on prior studies with mothers, to influence antibiotic use (Fuertes et al., 2022a, 2022b).

## 2. Materials and Methods

### 2.1. Sample

To minimize the confounding effect of variables known to affect infant or father behavior, the inclusion criteria are: (i) infants with no congenital malformations or pathology at birth; (ii) no time in the Neonatal Intensive Unit Care, and absence of disease or infirmity at the time of testing; (iii) fathers with no severe chronic medical, psychiatric conditions, or suspected alcohol/drug abuse according to medical records; (iv) the pregnancy should have been monitored and no major medical complications associated with delivery. Our sample was composed of 62 Portuguese infant and their fathers. Among infants, 36 were girls, 26 were boys, 39 were firstborns. Both parents lived in the same household as couples. Two fathers are of Brazilian nationality (but residents in Portugal), one Venezuelan, and the other fathers are Portuguese.

Half of the infants (31 out of 62, 50%) took antibiotics in the first year of life: three infants in the first three months, 14 between the 4th and 9th months, and 14 between the 10th and 12th months. Table 1 presents the demographic information of the sample comparing both groups (infants who took antibiotics in the first 12 months of life versus infants who did not). There are no significant differences between the two groups concerning paternal age, paternal years of formal education, number of father’s children, gestational weight of the baby, gestational age of the baby, Apgar 1st minute, and Apgar 5th minute.

### 2.2. Ethics

This study’s procedures were conducted according to the Declaration of Helsinki ethical guidelines. Accordingly, written informed consent was obtained from mothers and fathers for their own and their children’s participation before any assessment or data collection occurred. The Santarem Hospital Ethic Committee approved the study procedures, and the Faculty of Psychology Ethics and Deontology Committee approved this study (ref: 1.9/18 November 2021). Parents were invited to participate in a longitudinal study about the quality of parent–child attachment and relationships in the first year of infants’ lives. The methods of collection and conditions of participation were explained to parents. To avoid contamination effects on the participants’ behavior, parents were not informed of the study hypotheses (e.g., the hypothesis that infants’ difficult behavior or ambivalent attachment can be associated with antibiotic use).

Our data are available at https://osf.io/5c9k7, (accessed on 1 September 2024).

### 2.3. Procedures

#### 2.3.1. Recruitment

Health care professionals assessed the eligibility of the newborns and their parents. When they were found to be eligible, the families were contacted and asked by the health professional if they would be interested in participating in the study. Parents who responded affirmatively were then contacted by a female research assistant. Over two years, the research assistant contacted potential participants in the Maternity of Santarem Hospital when infants were born and explained the study’s purpose and procedures.

We determined the minimum sample size required to test this using G*Power version 3.1.9.7 (Faul et al., 2007). Results indicated that the required sample size to achieve 80% power for detecting a medium effect of d = 0.7, at significance level of α = 0.05, was N = 52 for *t*-test. All eligible parents of newborns delivered at that maternity hospital were contacted. Of the 85 eligible parents who agreed to participate in the study, 23 dropped out or lost their eligibility (moving from the district area of the study, infant serious illness, parents’ separation, etc.), resulting in a final sample of N = 62 fathers and infants.

#### 2.3.2. Data Collection

In this longitudinal study, data were collected at 3, 9, and 12 months. We used these three time-points to gather and update health and demographic information from both the infants and their fathers. At 3 months, we assessed the father–infant interaction quality for the first time, as previous research reports that these interactive behaviors observed at this age are predictors of later attachment (Barbosa et al., 2021). Attachment is generally assessed at 12 months, as attachment theory predicts that by this age, the bond between the infant and their attachment figures is established (Ainsworth et al., 1978). It was important to apply the stress and parental involvement scales after 9 months, a period when families have typically returned to work after maternity leave and have settled into stable routines.

#### 2.3.3. Fathers and Infants Demographic and Health Information

Fathers complete a demographic and health questionnaire at birth, 3 months, 9 months, and 12 months. This questionnaire was previously validated and used (Fuertes et al., 2016). The information collected included infant gender, parent’s age, and education level, parents’ nationality and immigration status, parents’ professional status, parent’s marital status, maternal health history, history of infertility, neonatal indicators (e.g., gestational age, weight, Apgar at first and fifth minutes), breastfeeding, infant health history (i.e., medical appointments, diseases, medication, and hospitalizations), and infant daycare attendance. To ensure accurate data collection, we requested parents to specify the names of the medicines used, which allowed us to verify the types of medicines administered. Medical consultations and other health information were obtained from the baby’s book, a record where health professionals document all relevant information about neonatal measurements, such as gestational age, gestational weight, Apgar scores, as well as weight progression, head circumference, and general health information. This book is given to all newborns in Portugal at birth and kept by the parents. During each medical visit, these data are recorded by health professionals. Therefore, in addition to the parents’ input, we had access to this information documented by health professionals. Fifty percent of the infants in our sample received antibiotics during the first 12 months of life, which aligns with findings from a previous study on the Portuguese pediatric population. The prescribed antibiotic, amoxicillin—with or without clavulanic acid, was primarily used to treat otitis and respiratory tract infections.

#### 2.3.4. Fathers and Infants Interactive Behavior in Free Play at 3 Months

At infants’ 3 months, recruited fathers were recontacted to schedule a follow-up lab visit with their infant. Father–infant dyads were videotaped in a free-play interaction.

Using the Crittenden Child–Adult Relationship Experimental Index (CARE-Index), infant and father interactive behavior was observed and coded from videotaped free-play interactions. Following the guidelines outlined in the CARE-Index manual (Crittenden, 2003), each dyad was recorded engaging in a 5 min play interaction. Fathers were encouraged to interact with their infants as they typically would at home during this session.

The CARE-Index included three adult scales, namely: Sensitivity, Control, Unresponsiveness, and four infant scales, Cooperativity, Compliant-compulsive, Difficulty, and Passivity (Crittenden, 2003). Each of these scales (paternal and infant scales) were coded in terms of facial expressions, verbal expressions, position and body contact, affection, turn-taking contingencies, control, and choice of activity. CARE-index is a dyadic measure, meaning assessing each other’s behavior considers the interactive context and its influence on the other partner.

Infant and paternal behavior were scored by two trained, reliable and blind (against antibiotic use) coders. The obtained overall average ICCs were consistently high for the CARE-Index paternal and infant scales at 3 months (paternal scales: 0.78 for sensitivity, 0.76 for control, 0.71 for unresponsiveness; infant scales: 0.88 for cooperation, 0.71 for compliant-compulsive, 0.91 for difficulty, and 0.81 for passivity). The final scores for discrepant cases were discussed and agreed upon by conferencing. To establish inter-coder reliability, one independent, external, and blind coder coded 20% of the data (randomly selected).

#### 2.3.5. Parents’ Involvement in Caregiving Activities Scale

At 9 months, fathers reported their involvement in 14 typical childcare activities with the infant (e.g., feeding, bathing, walking in the park, providing health care) using the Parents’ Responsibility Scale (McBride & Mills, 1993)—Portuguese version (Lima, 2005). The scores of this Likert Scale vary from 1 (always mother), 2 (usually mother), 3 (both parents shared the task), 4 (usually father), and 5 (always father). Although mothers did not participate in this study, both parents completed the instrument together and indicated who had primary responsibility for each task, to minimize bias in this measurement, as required by the scale’s manual.

#### 2.3.6. Parental Stress Scale

The Parental Stress Scale (PSS) (Berry & Jones, 1995) was created in 1995 to capture both positive aspects (e.g., emotional benefits, personal development) and negative aspects of parenthood (e.g., demands on resources, feelings of stress). This scale may be applied to mothers or fathers; in this study we applied the scale only to fathers. PSS is a brief and easy-to-administer self-report. It is an 18-item scale on which fathers respond to statements about their typical relationship with their child. For each statement, respondents rate their level of agreement on a 5-point Likert-type scale (1 = strongly disagree, 2 = disagree, 3 = undecided, 4 = agree, and 5 = strongly agree). To compute the paternal stress score, the positive Items 1, 2, 5, 6, 7, 8, 17, and 18 are reverse scored, and then, all items are summed. Higher scores reflect more paternal stress. The possible range of the PSS is 18 (low stress) to 90 (high stress). The scores of the scale were reliable, with a coefficient α of 0.83 and a mean interitem correlation of 0.23. A 6-week test–retest correlation was 0.81 (Berry & Jones, 1995); the Portuguese adaptation of the scale obtained good scores for global internal consistency (Algarvio et al., 2018). In our study, a report of paternal stress was collected at the 12-month visit.

#### 2.3.7. Father–Infant Attachment

At the 12-month visit, father–infant dyads were videotaped during the Strange Situation paradigm (SS) as developed by (Ainsworth et al., 1978). The SSP is a 21 min laboratory paradigm consisting of a sequence of eight episodes designed to place mild but increasing levels of stress on the infant and father–infant dyad (i.e., being introduced to an unfamiliar playroom, interacting with an unfamiliar adult stranger, and brief separations from and reunions with the father).

Videotapes of infants’ attachment behavior during the SS were scored by two independent, blind against antibiotic use, trained, and reliable coders following the procedures developed by (Ainsworth et al., 1978) and (Main & Solomon, 1990). Infants were classified as either securely attached (B), insecure-avoidant (A), and insecure-ambivalent/resistant (C). Intercoder reliability was good. The Cohen’s kappa coefficient for ABC classification was 0.80. All cases were double-coded. Discrepant cases were discussed and agreed upon by conferencing.

### 2.4. Analytic Plan

Descriptive statistics for the sample’s characteristics and other study variables were obtained using univariate statistics (sample size, mean and standard deviation). The Shapiro–Wilk test was used to evaluate the normality of the data; if *p* > 0.05, parametric tests were conducted. Analyses for Aim 1 and 3 utilized descriptive statistics and Student’s *t*-tests to evaluate the differences between the two study groups according to independent variables. Also, for Aim 3, we conducted Fisher’s Exact Test, followed by the determination of the effect size (Phi), the odd’s ratio, and the respective confidence intervals, to determine whether there was an association between antibiotic use and the type of daycare. To test if there is an association between antibiotic use and infant–father attachment pattern (Aim 2), we conducted a Pearson’s chi-square test. For Aim 4, we conducted a binary logistic regression (a forward stepwise (Wald) analysis) to identify the predictors influencing antibiotic use. We also determined the odd’s ratio, the respective confidence interval, and the Nagelkerke pseudo-R^2^ that adjusts the Cox and Snell pseudo-R^2^, to allow for a maximum value of 1.

## 3. Results

### 3.1. Aim 1—Differences in Fathers’ and Infants’ Interactive Behavior at 3 Months According to Antibiotic Use

In Table 2, we compare scores between dyads in which infants used antibiotics and those in which they did not. At 3 months, infants who took antibiotics are less cooperative (*M* = 7.77, *SD* = 2.046, versus *M =* 9.29, *SD* = 1.510), *t*(53.312) = −3.301, *p* = 0.002, CI_95%_ (mean difference) = [−2.449, −0.598], more difficult (*M* = 3.93, *SD* = 3.290, versus *M =* 1.10, *SD* = 1.795), *t*(44.550) = 4.161, *p* = 0.000, CI_95%_ (mean difference) = [1.463, 4.210], and less passive (*M* = 1.23, *SD* = 1.851, versus *M =* 2.74, *SD* = 2.049), *t*(58.729) = −3.019, *p* = 0.004, CI_95%_ (mean difference) = [−2.508, −0.509]. Hedges’ g to measure the effect size between the two groups were *g* = −0.839, CI_95%_ [−1.354, −0.317] for infant cooperativity, *g* = 1.062, CI_95%_ [0.526, 1.589] for infant difficulty, and *g* = −0.762, CI_95%_ [−1.273, −0.245] for infant passivity. In the three cases, *g* values suggest that the three factors have a large effect on antibiotic use (Table 2).

We also measured paternal interactive behavior according to three styles: sensitivity, control, and passivity. Results indicated a marginal (but not significant) lower sensitivity of fathers of infants who took antibiotics in the first 12 months than fathers of infants who did not (*M =* 8.37, *SD =* 2.297 versus *M* = 9.32, *SD* = 1.833), *t*(55.414) = −1.793, *p* = 0.078, CI_95%_ (mean difference) = [−2.024, 0.112]. The effect size is medium, as indicated by the Hedges’ *g* = −0.455, but with CI_95%_ [−0.955, 0.49], thus including the possibility of no effect (Table 2).

Similarly, fathers of infants who took antibiotics exhibited higher control interactive behavior than the fathers of infants that did not take antibiotics (*M =* 3.27, *SD =* 3.290 versus *M* = 1.77, *SD* = 2.591). The difference between the two groups was marginally significant, *t*(55.081) = 1.964, *p* = 0.055, CI_95%_ (mean difference) = [−0.030, 3.015]. The effect size is medium, as indicated by the Hedges’ g value of 0.499, but with the CI_95%_ [−0.007, 1.000], which includes zero. Paternal passivity did not differ between the two groups of fathers, [*t*(58.142) = −0.910, *p* = 0.366] (Table 2).

### 3.2. Aim 2—Antibiotic Use in the First Year of Life and Attachment Style at 12 Months

The prevalence of antibiotic use during the first 12 months of life and father–infant according to the attachment patterns was tested. The number of infants that took antibiotics did not differ according to their attachment pattern [χ^2^(2, *N* = 61) = 1.347, *p* = 0.510] (Supplementary Table S1). One infant did not exhibit a typical A, B, and C attachment according to Ainsworth Scales (Ainsworth et al., 1978), showing A strategy in the first reunion of Strange Situation and C in the second. Therefore, the total number of cases in this analysis is 61.

### 3.3. Aim 3—Exploring Possible Contributors to Antibiotic Use in Infancy

#### 3.3.1. Differences in Fathers’ Involvement in Health Care at 9 Months According to Antibiotic Use

To study putative associations between fathers’ involvement in their infants’ health care and infant consumption of antibiotics, both parents were asked to specify who had primary responsibility for each task (see Section 2). A total of 2 fathers and 13 mothers are usually responsible for their infant’s primary health care. A total of 46 of the 62 fathers offer primary health care to their infants with mothers, but none of the 62 fathers is the unique provider of primary health care to their infants (Table 3). In the case of medical appointments in the first 9 months of infants’ life, the trend is similar: no father and 10 mothers are usually responsible for infants’ medical appointments attendance, but 51 fathers share this task with mothers. The mother of one of the infants is solely responsible for bringing the child to the medical appointment (Table 3). When the baby is unwell, 2 mothers are always responsible for caring for the infant, 20 mothers and 4 fathers are usually responsible, and 36 fathers share the responsibility with mothers (Table 3).

No association was found between fathers’ involvement in their infants’ health care and infant consumption of antibiotics (Supplementary Table S2).

#### 3.3.2. Associations Between Paternal Stress Reported When Infants Were 12 Months and Antibiotic Use

To address this point, we administered a self-report questionnaire on paternal stress. To avoid Type I errors due to conducting multiple comparisons, we adjusted the significance threshold with a Bonferroni correction, α′ = 0.00278. Thus, the stress levels of fathers of infants who took antibiotics did not differ from those of fathers of infants that did not take antibiotics (Supplementary Table S3).

These findings suggest that the stress levels of the group of fathers of infants who did not take antibiotics do not differ from those who did.

#### 3.3.3. Daycare Attendance According to Infants’ Antibiotic Use During the First Year of Life

In Portugal, center-based daycare attendance starts at 4 months of age. In our study, 60 infants attended center-based or family-based daycare between 6 and 12 months. Two families did not inform us about the type of daycare their infants attended.

Most infants (29/30 = 97%) who received antibiotics attended center-based daycare at 12 months of age, while infants who did not were more evenly distributed between attending center-based daycare (19/30 = 63.3%) and family-based daycare (11/30 = 36.7%). Conversely, just one infant among 12 attending family-based daycare took antibiotics (1/12 = 8.3%), whereas 29/48 = 60.4% of the infants attending center-based daycare took antibiotics (Table 4). Fisher’s Exact Test was conducted to determine whether there was an association between antibiotic use and the type of daycare (center-based vs. family-based). Fisher’s Exact Test revealed that the association was significant, *p* = 0.002, suggesting that infants attending center-based daycare were more likely to use antibiotics. The effect size, measured by the Phi coefficient, was Φ = 0.41, indicating a moderate association. The odds of attending family-based care are 0.06 as likely for children who took antibiotics CI_95%_ (odds ratio) = [0.007, 0.5], indicating that infants who took antibiotics were much less likely to attend family-based care. Conversely, the odds of attending family-based care are 16.8 times higher for children who did not take antibiotics, with CI_95%_ (odds ratio) = [2, 143].

### 3.4. Aim 4—Determinants of Antibiotic Prescription

We found differences in behavior of infants who received antibiotics in the first 12 months of life from those who did not: infants who took antibiotics are less cooperative, more difficult, and less passive and are likely to attend a center-based daycare at 12 months. Using these four significant variables associated with antibiotic use, we conducted a binary logistic regression (a forward stepwise (Wald) analysis) to identify the predictors influencing antibiotic use (Table 5). In the first step, Infant Difficultness at 3 months was found to be a predictor, with a positive association with antibiotic use (*β* = 0.394, Wald = 11.154, *p* = 0.001). The odds ratio for Infant Difficultness is e^β^ = 1.483, which is higher than 1, and with a 95% confidence interval CI_95%_ = [1.177, 1.868] that does not include the value of 1. This means that for every one-unit increase in Infant Difficultness, the odds of antibiotic use increase by approximately 48.3%. The likelihood of the observed data under the model, -2 Log Likelihood value was 67.027. The Nagelkerke R^2^, a pseudo-R^2^ (that adjusts the Cox and Snell R^2^, another pseudo-R^2^, to allow for a maximum value of 1) was 0.295 (Supplementary Table S4 presents the variables not included in the binary logistic regression).

In the second step, Infant Difficultness (*β* = 0.347, Wald = 7.94, *p* = 0.005) and Daycare Attendance (*β* = −2.379, Wald = 4.423, *p* = 0.035) were identified as predictors of antibiotic use. The odds ratio for Infant Difficultness is e^β^ = 1.415, with a 95% confidence interval CI_95%_ = [1.111, 1.801], which does not include the value of one. This suggests that, after controlling for Daycare Attendance, each one-point increase in the Infant Difficultness score is associated with a 41.5% rise in the odds of using antibiotics. Additionally, in the second step of the model, Daycare had an odds ratio of e^β^ = 0.093, where *β* = −2.379 is the Wald, with CI_95%_ = [0.01, 0.85], which does not include the value 1. An odds ratio of 0.093 indicates that, while holding the Infant Difficultness predictor constant, the odds of antibiotic use decreases by 90.7% (1 − 0.093 = 90.7%) when infants attend family-based daycare. The −2 Log Likelihood value was 60.374, lower than in the first step, indicating a better fit of the model to the data. The Nagelkerke R^2^ was 0.406, “explaining” 40.6% of the variability of antibiotic use and indicating a moderate fit of the model to the data (Table 5).

## 4. Discussion

Antibiotic stewardship has proven intricate as social, cultural, and psychological factors linked to antibiotic overuse have been identified (Calvo-Villamañán et al., 2022; Deschepper et al., 2008; Dionisio et al., 2023b; Grigoryan et al., 2008; Krockow et al., 2022). From the psychological factors identified, previous research has shown that maternal sensitivity, infant cooperative and difficult behavior, and infant–mother attachment patterns are associated with antibiotic use in the first months of infants’ lives (Fuertes et al., 2023b). Following this line of research, we included fathers in our research, aiming to learn about their role in antibiotic use in infancy.

Attachment Theory, proposed by John Bowlby (1969), states that caregiver behaviors significantly impact the organization of attachment and the overall well-being of the infant (Bowlby, 1969). Contributing to this body of knowledge, we found that a father’s ability to respond interactively to an infant’s needs (paternal sensitivity and paternal control) is not associated with antibiotic use. These results contrast with previous research that found maternal sensitivity was a predictor of infants’ use of antibiotics in infants born full and preterm (Fuertes et al., 2023b, 2023a). One possible explanation for this discrepancy is that paternal sensitivity scores tend to be lower and less variable than maternal sensitivity scores, which may reduce statistical power to detect associations. Previous research has reported an average maternal sensitivity of approximately 9.92. (Barbosa et al., 2021)—even when including families at social and educational risk, who were not part of our sample—compared to a paternal sensitivity of 8.85 across all fathers in this study. Indeed, comparative Portuguese studies have shown that mothers tend to be more sensitive than fathers, and their infants are more likely to form secure attachments to them (Fuertes et al., 2016). Moreover, while both more sensitive mothers and fathers tend to be more involved in daily caregiving routines, fathers typically perform fewer caregiving tasks and dedicate less time to infant care (Fuertes et al., 2016). Acknowledging the differences between maternal and paternal caregiving in Portugal is essential to understanding these results. Portuguese legislation has ensured paternal leave rights since 1999, which has been successively increased to 30 mandatory minimum days and the same rights as mothers to stay with their children when sick. However, mothers have 6 months of maternal leave, and the adherence of working Portuguese fathers in sharing parents is only 45.7% (Fernandes et al., 2023). In sum, more comparative studies are necessary to discriminate paternal and maternal interactive behavior impact on antibiotic use during infancy.

However, it is not only parents’ behavior that accounts for antibiotic uptake. As in previous studies conducted with mothers, we found that infants who took antibiotics exhibited more difficult behavior with fathers and were less cooperative during free-play interactions compared to other infants (Fuertes et al., 2023a, 2023b). First, as previous research has found, infants with fussier and more externalizing behavior tend to somatize their health symptoms of infectious diseases. Another explanation is that infants’ protests (crying, reactivity, etc.) alert their parents, while more passive or cooperative behaviors worry less about their parents. Worried parents may expect medication to ameliorate the infants’ health condition. Corroborating this explanation, one study has shown that when the diagnosis is uncertain, or infants are stressed, mothers wish for antibiotics to speed up their children’s recovery (Bosley et al., 2022). The mothers in that study either had little knowledge of the problems associated with antibiotic use or devalued the adverse effects of these drugs.

Furthermore, we tested the prevalence of antibiotic use according to infant attachment patterns to their father at 12 months. Our study shows that infant–father attachment patterns are not associated with antibiotic use. Nonetheless, previous studies have shown that infants insecurely ambivalently attached to their mothers were more likely to take antibiotics in the first year of life (Fuertes et al., 2023a, 2023b). It would be very interesting to conduct a future study to verify the infant attachment pattern with both the mother and father and test the differences in the prevalence of antibiotic use according to the infant’s attachment pattern. In fact, each relationship is unique. Since birth, infants react to their partners’ social and emotional stimulus (e.g., their parent’s facial expression, tone of voice, touch); parents read these responses and help infants to regulate their behavior; with time, infants learn to self-regulate and coregulate interactions (Tronick et al., 2020). Relationships result from transactional processes that lead to independent developmental and emotional paths (Madigan et al., 2024), which seems to be the case with antibiotic use and attachment between mothers and fathers. Hence, the findings of this study corroborate the hypothesis that infant–mother and infant–father relationships have different impacts on child development, well-being, and health, and, in this case, on the use of antibiotics.

Beyond the socioemotional factors, we reason that paternal stress and involvement in health care might affect their perspective on infant illness. Previous studies have shown that working-age patients are more likely to receive inappropriate antibiotic prescriptions (e.g., infections caused by viruses) (Dekker et al., 2015; Denny et al., 2019). Therefore, we hypothesize that when working fathers care for their ill infants, they may feel pressured to return to their work responsibilities. In this scenario, fathers may desire their infants to recover quickly and may unconsciously pressure general practitioners to prescribe antibiotics. To test this hypothesis, further qualitative studies should explore fathers’ motivations and perceptions regarding antibiotic use. Nevertheless, our results describe fathers as individuals who approach fatherhood with little stress, and develop positive interactions with their infants, rarely perform care activities alone, and instead share care with mothers. Our findings align with a previous study indicating that fathers experience lower stress levels than mothers (Malmberg & Flouri, 2011).

Past Portuguese comparative studies have found that mothers are significantly more involved in caregiving than fathers (Fuertes et al., 2016). However, in the current sample, collected ten years later, the gap between maternal and paternal involvement appears to be smaller. In this sample, mothers provided care either alone or jointly with fathers. Notably, health care-related tasks were among the caregiving domains in which fathers were most frequently involved. However, although 74% (46/62) of fathers offer primary health care to their infants, none of the 62 fathers is the unique provider of primary health care to their infants. That may explain why we did not find an association between infants’ use of antibiotics and fathers’ involvement in infants’ health care.

Of course, the exposition of infectious agents needs to be considered as a possible factor; therefore, we studied the prevalence of antibiotic use according to daycare attendance. It is expected that contamination of bacterial diseases will be higher in an environment with 12 infants per room, as is the case in Portuguese nurseries (center-based daycare), especially when they start crawling. Our results corroborate this hypothesis. Thus, educational authorities should properly regulate daycare centers to decrease contagion among infants (for instance, having isolation rooms for children to wait for their parents to pick them up when with fever). Center-based daycare attendance is associated with a higher risk of infectious diseases among children. For example, a study conducted in France involving 1263 children aged 3 months to 3 years revealed that the risk of developing the first episode of otitis, common cold with fever, and wheezy bronchitis was significantly higher in children who had recently started attending daycare centers compared to those who stayed at home (Collet et al., 1991). Although some of these infections are viral and self-limiting, sometimes antibiotics are prescribed (Dekker et al., 2015; Pouwels et al., 2018).

The main objective of our study was to identify predictors of antibiotic use among psychological and health factors. Two predictors of antibiotic use were found in this sample. The first and strongest is the infant’s difficult behavior, as found in previous research with mothers (Fuertes et al., 2022a, 2022b). The second is the frequency of daycare centers between 6 and 12 months. If the second is expected, the first is nouvelle information that deserves careful attention. Previous studies have shown that various psychological factors contribute to antibiotic prescription, both from the perspectives of patients and health professionals. Pediatric visits present a higher level of complexity because, in addition to the health professional and the patient, there is often a third party involved: typically, the father or the mother. This fact presents a new level of complexity to this problem: finding the psychological factors leading to overprescribing antibiotics.

Last, these findings may help avoid unnecessary antibiotic use, which is crucial for both families and health care professionals. We have shown that infant behavior (including cooperativeness, difficulty, and passivity) is associated with antibiotic use. Therefore, health care professionals should consider this factor when assessing the need for antibiotic prescriptions, such as by inquiring with parents (e.g., type and duration of symptoms, or reactions of the infant to sickness) or through additional clinical tests. This is important because overuse and misuse of antibiotics can lead to antibiotic resistance, making infections harder to treat and increasing the risk of severe illness and death. They can also disrupt the natural balance of bacteria in the body, causing side effects like gastrointestinal issues and allergic reactions. By avoiding unnecessary antibiotic use, we can protect public health, ensure effective treatments remain available, and reduce the burden of antibiotic resistance on health care systems. Preventive actions appear to be more effective in the long term. One important recommendation is the inclusion of isolation rooms in Portuguese daycare centers for children presenting with fever or other symptoms of infectious illness. Currently, in most daycare settings in Portugal, when a child shows symptoms of illness, parents are notified and expected to collect the child immediately, even though the child may remain in contact with others until pickup. Instead of requiring parents to leave work immediately, isolation rooms—for less severe cases, such as low-grade fevers—could allow families to balance professional responsibilities with caregiving demands, while simultaneously preventing the spread of illness to other children. Additionally, schools in Portugal, which may be attended by hundreds of children, should be equipped with full-time health care professionals to ensure appropriate health monitoring and early intervention. These professionals can advise and assure parents before they attend medical consultations, reducing parental stress and disinformation.

### Studies Limitations, Strengths, and Future Directions

Regarding the study limitation, this is a very low biological and social risk sample; for instance, fathers had well-paid professions and higher levels of education compared to the Portuguese population. The characteristics of this sample may limit the generalizability of the findings. Another limitation is the laboratory-based nature of the father–infant interaction assessments. Although most studies have used such measures, facilitating comparisons across findings, observations conducted in naturalistic settings may better capture the spontaneity, contextual nuances, and ecological validity of everyday father–infant interactions. Therefore, future research should also include more diverse participants and measures.

Also, it is important to include couples to learn more about each parent’s impact on infant antibiotic consumption. Future studies should include both parents to allow for a comprehensive understanding of each parent’s contribution to decisions regarding infant health. This approach would also enable a more thorough analysis of their individual perspectives on antibiotic use, medication in general, and broader attitudes toward their child’s well-being.

Despite the limitations, this study is novel in including fathers (a key caregiver that is less studied than mothers) and deepens our knowledge of the causes of infant excessive use of antibiotics in the first year of life. These findings also contribute to Attachment Theory by demonstrating how early relational experiences—reflected in infant behavior and parental functioning under stress—may influence not only socio-emotional outcomes, as traditionally emphasized, but also health-related behaviors such as antibiotic use.

## 5. Conclusions

Infants who were given antibiotics in their first year were less cooperative, more difficult, and less passive during free play with their fathers. They were also more likely to attend center-based daycare. Their fathers displayed slightly lower sensitivity and more controlling behaviors than fathers of infants that did not take antibiotics. The main predictors of antibiotic use included infant difficulty and the type of daycare (family-based versus center-based. In comparison to previous studies involving mothers, paternal behavior had a weaker association with antibiotic use.

The findings suggest the potential value of early parental guidance programs aimed at emotional regulation in infants and reducing unnecessary medical interventions.

## Figures and Tables

**Table 1:** Demographic information of the sample comparing the two groups: infants who took antibiotics in the first 12 months of life versus the others.

	Antibiotic Use (12 Months)	N	M	SD	t	*p*	Hedge’s g
Paternal age	Yes	31	35.0	5.86	−0.10	0.92	−0.02
	No	31	35.1	7.01			
Paternal years of formal education	Yes	31	12.1	2.74	−1.34	0.97	−0.34
	No	31	13.1	2.92			
Nr of fathers’ children	Yes	31	0.35	0.55	−0.99	0.33	−0.25
	No	31	0.52	0.72			
Gestational age of the baby (weeks)	Yes	31	39.5	1.03	0.85	0.40	0.21
	No	31	39.3	1.07			
Birthweight of the baby (g)	Yes	31	3271	423	−0.42	0.68	−0.10
	No	31	3315	399			
Apgar at 1st minute	Yes	31	9.19	0.79	0.71	0.48	0.18
	No	31	9.06	0.63			
Apgar at 5th minute	Yes	31	9.94	0.25	0.72	0.47	0.18
	No	31	9.87	0.43			

**Table 2:** Fathers’ and infants’ interactive behavior assessed with the Care-index scale at 3 months according to antibiotic use: Means, Standard Deviations, *t*-tests, effect size (Hedges’ g), and CI_95%_.

					*t*-Test for Equality of Means					Effect Size of Difference		
								CI_95%_ of Difference			CI_95%_ of g	
	Took Antibiotics	N	M	SD	t	*p*	Mean Difference	Lower	Upper	Hedges’ g	Lower	Upper
Infant cooperation	Yes	30	7.77	2.05	−3.301	0.002	−1.524	−2.449	−0.598	−0.839	−1.354	−0.317
	No	31	9.29	1.51								
Infant compulsion	Yes	30	1.07	2.35	0.649	0.519	0.357	−0.745	1.459	0.165	−0.332	0.660
	No	31	0.71	1.92								
Infant difficulty	Yes	30	3.93	3.29	4.161	0	2.837	1.463	4.21	1.062	0.526	1.589
	No	31	1.1	1.8								
Infant passivity	Yes	30	1.23	1.85	−3.019	0.004	−1.509	−2.508	−0.509	−0.762	−1.273	−0.245
	No	31	2.74	2.05								
Paternal sensitivity	Yes	30	8.37	2.3	−1.793	0.078	−0.956	−2.024	0.112	−0.455	−0.955	0.049
	No	31	9.32	1.83								
Paternal control	Yes	30	3.27	3.29	1.964	0.055	1.492	−0.03	3.015	0.499	−0.007	1.000
	No	31	1.77	2.59								
Paternal passivity	Yes	30	2.37	2.4	−0.91	0.366	−0.537	−1.717	0.643	−0.230	−0.727	0.268
	No	31	2.9	2.2								

**Table 3:** Parental involvement in health care at 9 months descriptive statistics.

	Responsibility in Primary Health Care		Taking the Infant to Doctor When Sick		Caring for the Infant When Sick	
	Frequency	Percentage	Frequency	Percentage	Frequency	Percentage
Always the mother	1	1.6	1	1.6	2	3.2
Typically the mother	13	21	10	16.1	20	32.3
Both parents	46	74.2	51	82.3	36	58.1
Typically the father	2	3.2	0	0	4	6.5
Total	62	100	62	100	62	100

**Table 4:** Associations between daycare attendance at 12 months and Infant–Father Attachment Pattern: Fisher’s Exact Test, effect size Φ, odds ratio and its CI_95%_ *.

Took Antibiotics	Center-Based	Family-Based	Total
No	19	11	30
Yes	29	1	30
Total	48	12	60

* *p* = 0.002, Φ = 0.41, moderate association, OR = 0.06 with CI_95%_ = [0.007, 0.5].

**Table 5:** Summary of binary logistic regression analyses predicting antibiotic’s prescription.

		β	S.E.	Wald	df	Sig.	e^β^	CI_95%_ for e^β^	
								Lower	Upper
Step 1 ^a^	Infant Difficulty	0.394	0.118	11.154	1	<0.001	1.483	1.177	1.868
	Constant	−0.96	0.385	6.208	1	0.013	0.383		
Step 2 ^b^	Infant Difficulty	0.347	0.123	7.94	1	0.005	1.415	1.111	1.801
	Daycare Attendance	−2.379	1.131	4.423	1	0.035	0.093	0.01	0.85
	Constant	1.879	1.298	2.096	1	0.148	6.546		

a. Variable entered on step 1: Infant Difficulty; b. Variable entered on step 2: Daycare Attendance.

## Data Availability

Our data are available at https://osf.io/5c9k7 without information that could identify participants.

## Supplementary Materials

The following supporting information can be downloaded at: https://www.mdpi.com/article/10.3390/ejihpe15050066/s1, Table S1: Associations between Antibiotic Use at 12 Months and Infant–Father Attachment Pattern: Pearson’s χ^2^-test; Table S2: Parental involvement in health care at 9 months descriptive statistics; Table S3: Paternal Stress and Antibiotic Use; Table S4: Variables not in the Equation in each step of the binary logistic regression analyses.

## Abbreviations

The following abbreviations are used in this manuscript:

CI_95%_	Confidence Interval 95%
M	Mean
SD	Standard Deviation
OR	Odds Ratio
