# Supplementary material for: Health and Psychological Predictors of Antibiotic Use in Infancy and Fathers’ Role
Source: Eur J Investig Health Psychol Educ. 2025 Apr 25;15(5):66. doi: 10.3390/ejihpe15050066 (PMC12111352; doi:10.3390/ejihpe15050066)
Supplement: Supplementary file 1 [file ejihpe-15-00066-s001.zip › ejihpe-3555962-supplementary.pdf]

SUPPLEMENTARY TABLES OF:

Health and Psychological Predictors of Antibiotic Use in Infancy and Fathers' Role

Table S1. Associations between Antibiotic Use at 12 Months and Infant- Father Attachment Pattern: Pearson's χ^2-test

	Infant-father attachment pattern			
Took antibiotics	A	B	C	Total
No	8	14	8	30
Yes	10	10	11	31
Total	18	24	19	61

$\chi^2(2, N = 61) = 1.347, p = .510$

Table S2 Parental Responsibility at 9 months and Antibiotic use for 12 months: Means, Standard Deviations, t-tests, mean differences and the respective confidence intervals (95%).

					t-test for Equality of Means				
								$CI_{95\%}$ of the Difference	
	Took antibiotics	N	M	SD	t	p	Mean Diff.	Lower	Upper
Responsibility in Primary Health Care	Yes	31	2.8	0.60	0.244	0.8	0.03	-0.23	0.30
	No	31	2.8	0.43					
Taking the infant to doctor when sick	Yes	31	2.8	0.40	0.000	1.0	0.00	-0.22	0.22
	No	31	2.8	0.48					
Caring for the infant when sick	Yes	31	4.1	1.73	-0.932	0.4	-0.35	-1.12	0.41
	No	31	3.5	1.71					

Table S3 Paternal Stress and Antibiotic Use

Question*	Took Antibiotics	N	M	SD	t-test for Equality of Means: t	p	Mean Difference	CI95% Lower	CI95% Upper
I am happy in my role as a parent	Yes	30	4.8	0.75	-0.950	0.349	-0.133	-0.419	0.152
	No	30	5.0	0.18					
There is little or nothing I wouldn't do for my infants if it was necessary.	Yes	30	4.6	1.22	-1.027	0.310	-0.267	-0.789	0.256
	No	30	4.9	0.73					
Caring for my infants sometimes takes more time and energy than I have to give	Yes	30	3.3	1.39	-0.398	0.692	-0.133	-0.804	0.538
	No	30	3.4	1.19					
I sometimes worry whether I am doing enough for my infants.	Yes	30	3.6	0.96	0.632	0.530	0.167	-0.361	0.694
	No	30	3.5	1.07					
I feel close to my infants.	Yes	30	4.8	0.38	0.297	0.768	0.033	-0.192	0.258
	No	30	4.8	0.48					
I enjoy spending time with my infants.	Yes	30	4.9	0.25	-1.439	0.161	-0.067	-0.161	0.028
	No	30	5.0	0.00					
My infant is an important source of affection for me.	Yes	30	4.9	0.25	-0.584	0.562	-0.033	-0.148	0.081
	No	30	5.0	0.18					
Having infants gives me a more certain and optimistic view for the future.	Yes	30	4.7	0.52	0.857	0.395	0.133	-0.179	0.445
	No	30	4.6	0.67					
The major source of stress in my life is my infants.	Yes	30	1.8	1.10	1.329	0.191	0.300	-0.155	0.755
	No	30	1.5	0.57					
Having infants leaves little time and flexibility in my life.	Yes	30	2.3	1.01	0.261	0.795	0.067	-0.444	0.578
	No	30	2.2	0.96					
Having infants has been a financial burden.	Yes	30	2.7	1.23	0.947	0.347	0.300	-0.334	0.934
	No	30	2.4	1.22					
It is difficult to balance different responsibilities because of my infants.	Yes	30	1.8	0.79	-1.884	0.065	-0.433	-0.894	0.028
	No	30	2.3	0.98					
The behavior of my infants is often embarrassing or stressful to me.	Yes	30	1.3	0.53	-2.599	0.012	-0.400	-0.708	-0.092
	No	30	1.7	0.65					
If I had it to do over again, I might decide not to have infants.	Yes	30	1.2	0.76	1.166	0.252	0.167	-0.124	0.458
	No	30	1.0	0.18					
I feel overwhelmed by the responsibility of being a parent.	Yes	30	1.4	0.56	-0.198	0.843	-0.033	-0.370	0.303
	No	30	1.4	0.73					
Having infants has meant having too few choices and too little control over my life	Yes	27	1.5	0.58	0.116	0.908	0.019	-0.303	0.340
	No	30	1.5	0.63					
I am satisfied as a parent	Yes	30	4.9	0.25	0.000	1.000	0.000	-0.131	0.131
	No	30	4.9	0.25					
I find my infants enjoyable	Yes	30	5.0	0.18	-1.000	0.326	-0.033	-0.102	0.035
	No	30	5.0	0.00					

*Questions asked to fathers when infants were 12 months old.

Table S4 Variables not in the Equation in each step of the binary logistic regression analyses

			Score	df	*p*
Step 1	Variables	Infant Cooperation	0.845	1	0.358
		Infant Passivity	0.265	1	0.607
		Daycare attendance	5.810	1	0.016
	Overall Statistics		8.069	3	0.045
Step 2	Variables	Infant Cooperation	1.275	1	0.259
		Infant Passivity	0.932	1	0.334
	Overall Statistics		2.694	2	0.260
